# Locally induced Stark shifts of collective excitonic modes in polyradical aggregates

**DOI:** 10.3762/bjnano.17.81

**Published:** 2026-08-25

**Authors:** Amandeep Sagwal, Rodrigo Cezar de Campos Ferreira, Petr Kahan, Maximilian Rödel, Jindřich Nejedlý, Jiří Doležal, Martin Švec

**Affiliations:** 1 Institute of Physics, Czech Academy of Sciences, Cukrovarnická 10/112, Praha 6 CZ16200, Czech Republic; 2 Faculty of Mathematics and Physics, Charles University, Ke Karlovu 3, CZ12116 Praha 2, Czech Republic; 3 Institute of Organic Chemistry and Biochemistry, Czech Academy of Sciences, Flemingovo náměstí 542/2, Praha 6 CZ16000, Czech Republic; 4 Faculty of Nuclear Sciences and Physical Engineering, Czech Technical University in Prague, Břehová 7, 115 19 Prague, Czech Republic

**Keywords:** chromophore, dark states, exciton, Stark shift, TEPL, tip-enhanced photoluminescence

## Abstract

Active control of dark long-lived excitonic states in molecular aggregates using local electric fields is a pivotal challenge for advancing nanoscale optoelectronics and quantum device engineering. This experimental study investigates the collective excitonic states in aggregates composed of radical chromophores. With the strong optical enhancement provided by tip-enhanced photoluminescence spectroscopy, we observe fingerprints of bright and dark excitonic modes appearing due to interexciton coupling and induced changes in their spectra with the electric field locally applied within the nanocavity gap. The analysis of the Stark shifts revealed a divergent behavior of the bright states in asymmetric measurement positions of the nanocavity above the aggregates. The observed complex behavior is discussed considering the influence of the field, molecule arrangement, nanocavity coupling, and electrostatic charge inhomogeneities in the clusters. This sensitivity to the external parameters demonstrates an effective means of control over radical excitonic aggregates.

## Introduction

Aggregates of chromophores with prevailing coulombic coupling are known to host well-defined collective excitonic modes in contrast to systems with an admixture of charge-transfer interactions [1–5]. In such systems, superradiant modes may emerge, providing the advantage of enhanced radiative rates; also, the dark states with their characteristically long lifetimes hold the promise to support efficient energy transfer among chromophores, which can be of high importance for the development of future optoelectronic devices and may help to better understand fundamental energy harvesting mechanisms in living systems [6–8]. In contrast to the neutral chromophore aggregates that have been studied quite extensively in this context recently, reports on systems composed of radical anions are scarce [9–13], despite their potential to host more exotic excitonic properties. Since individual anion radicals often exhibit higher polarizability, they are prone to intermolecular static charge redistribution and screening, which can render them responsive to external fields, useful for excitonic control and switching [14]. Their open-shell character may also bring spin-dependent functionality, which can lead to higher fluorescence efficiency due to different relaxation pathways [15] and magnetic field-activity due to the unpaired electron [16–19]. To tackle the problem of creating and investigating such stable radical anion aggregates, one of the promising strategies is to precisely assemble well-defined clusters of precursors with strong acceptor or donor character on insulating crystalline surfaces.

Exploring the collective excitonic modes of aggregates requires specialized approaches that combine high spatial and spectral resolution under cryogenic conditions. The reason is the fine (Davydov) splitting and energy ordering of spectral fingerprints of the excitonic modes of various individual clusters [20–23]. This is beyond the reach of conventional optical spectroscopy working in the far field, hindered by the Abbe diffraction criterion, inhomogeneous broadening, validity of Kasha’s rule, and weak optical activity of dark modes due to the symmetry-imposed selection rules or forbidden electronic transitions [5,24]. In addition, achieving local control over the excitons requires a highly defined geometry and environment of the nanostructures. Emerging near-field spectroscopic techniques employ an extremely localized electromagnetic field confined in the junction of a scanning probe microscope to make the otherwise forbidden transitions optically accessible [25–29]. With this approach, demonstrations of the sensitivity to the dark states were performed on various neutral phthalocyanine assemblies on thin crystalline insulating layers with outstanding precision. It permitted investigation of their coherence and entanglement using scanning tunneling microscope-induced electroluminescence (STM-EL) [30–33]. In our previous work, we have applied STM-EL to investigate small aggregates of widely studied electron acceptors, perylene dicarboxylic anhydride (PTCDA), which can be stabilized in a dominantly radical state in the nanocavity, and revealed hints that its charge state could be controlled with local application of an electric field [34]. However, the STM-EL method requires the application of bias above a threshold to drive the excitations by injection of charges; consequently, it cannot track the excitonic response to a continuously tuned field in the nanocavity and evaluate Stark and vibronic shifts. This can be overcome by the tip-enhanced photoluminescence (TEPL) technique, which allows one to access the excitonic modes without the need for an external bias [35–37]. Notably, this approach remains currently underexploited.

In this work, we investigate the effect of the electric field on the photon response of dominantly anionic PTCDA aggregates confined in an STM plasmonic nanocavity at the submolecular level, focusing on the characteristic behavior of the bright and dark exciton modes. We confirm the controllability of the collective modes by the electric field, by the aggregate geometry, and by the nanocavity positioning in various clusters. We discuss the complex behavior of the bias-dependent TEPL spectra in the context of a simple model that allows us to estimate the Stark shifts of the individual chromophores, their mutual coulombic coupling, and the influence of the nanocavity.

## Results and Discussion

**Figure 1:**
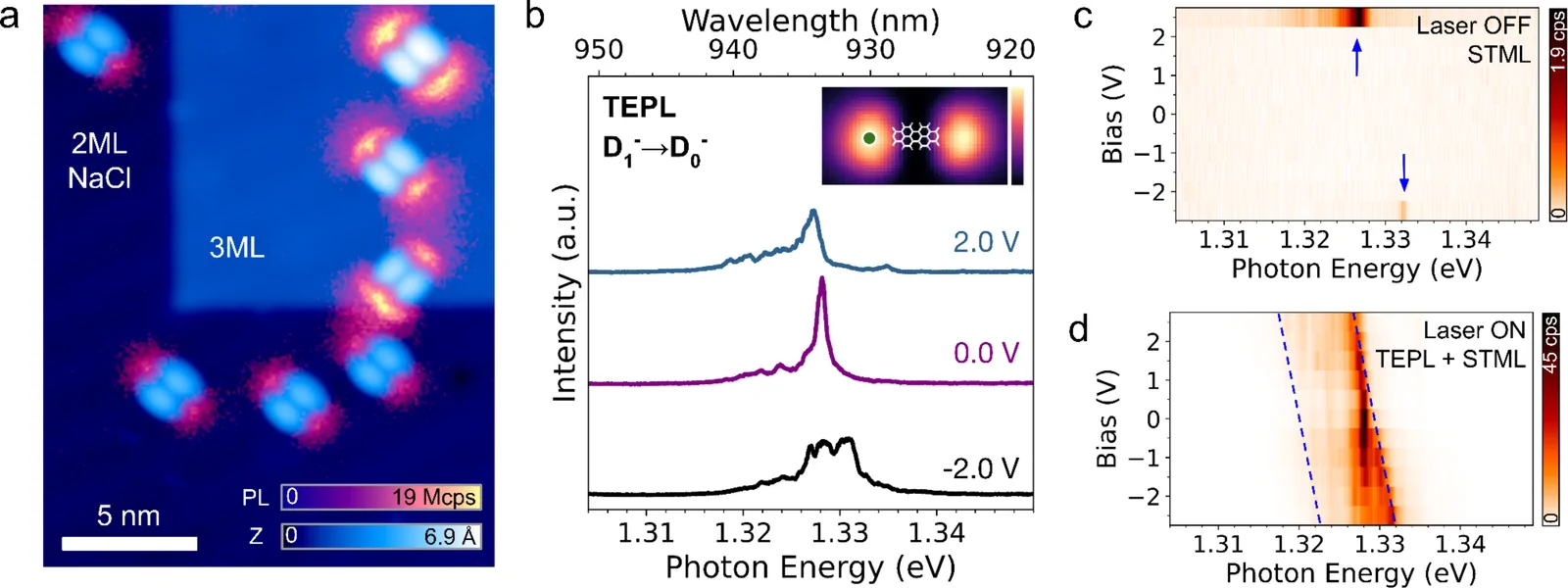
(a) STM constant-current topography and tip-enhanced luminescence of PTCDA on a 2–3 MLs NaCl/Ag(111) crystal surface. Scanning conditions: 900 mV, 3 pA, 16.9 × 23.8 nm^2^. The excitation wavelength of the laser was 785 nm (1.579 eV) with 1.1 mW total output power. The photon rate map was taken simultaneously using an avalanche photodiode with a 925/25nm filter and superimposed over the topographic image using a different color scale. (b) Representative TEPL spectra acquired at −2.0, 0.0, and 2.0 V. The inset shows a constant-height TEPL intensity map for the D_1_^−^ → D_0_^−^ radiative transition, shown for the 1.328 eV energy at zero bias. The green dot marks the location of the point-spectra measurements in (c) and (d). The size of the map is 4.8 × 2.9 nm^2^, 48 × 29 points. (c, d) Spectral intensity as a function of bias without and with laser excitation, respectively. The vertical arrows in (c) denote the onsets of the electroluminescence. The blue dashed line in (d) marks a common trend and high-energy cutoff of the bias-dependent TEPL spectra.

First, we studied bias-dependent spectra of an individual PTCDA molecule. A composite image of surface topography (*Z*) and tip-enhanced photoluminescence signal (TEPL) of individual PTCDA molecules adsorbed on 2 and 3 monolayers (MLs) of NaCl on Ag(111) is shown in Figure 1a. Above each of the molecules, a characteristic two-lobe luminescence pattern is observed. This pattern originates from the molecule’s radiative electronic transition from its first excited state to the ground state (D_1_^−^ → D_0_^−^), which is coupled to the nanocavity [14]. At 3 MLs, the luminescence yield is stronger compared to 2 MLs; it can be attributed to a better decoupling from the metal substrate and a more efficient radiative decay rate. Therefore, we will focus exclusively on molecules on 3 MLs in this study [38–41]. The point spectra of the D_1_^−^ → D_0_^−^ emission in Figure 1b, taken above the carbonyl terminations, are presented for three different bias values at −2.0, 0.0, and 2.0 V. Such strong variations among these spectra indicate an effect of the electric field in the tip–sample junction. With respect to the spectrum obtained at zero bias, which has one narrow dominant peak with small sidebands, the −2.0 and 2.0 V spectra show a general blue- and redshift of a few millielectronvolts, respectively, and the spectral envelope is notably widened for the spectrum at −2.0 V.

Detailed bias dependence measurements with and without illumination allow us to put the bias-induced Stark shift and complex restructuring of the spectral envelope into the context of the previously reported STM electroluminescence measurements [34,42–43]. The 2D heatmaps created from individual spectra in Figure 1c,d provide a comparison of the two scenarios on the same system. The electroluminescence (in Figure 1c) manifests an onset at −2.5 V, producing a very sharp peak at 1.332 eV. At the positive limit of 2.5 V, a broader electroluminescence peak is found at ≈5.4 meV lower energy. No measurable signal is detected between the onsets in this setup. In contrast, TEPL (Figure 1d) provides spectra for all biases in the range and reveals that the overall spectral envelope (delimited by the dashed lines) shifts constantly by approximately −0.9 meV/V. The electroluminescence and TEPL are overlapping at ±2.5 V with their most intense features; however, TEPL has a stronger overall yield and a different spectral lineshape than the electroluminescence. In addition, a closer look at the spectra in the entire bias range reveals transient sharp spectral components within the broad envelope that barely change energy with the applied bias. The most intense is a prominent peak occurring at 1.328 eV in the range from −1.0 to 1.0 V. We attribute these peaks tentatively to the effects of resonant Raman scattering that may match vibronic transitions, as it was suggested in a previous study of single phthalocyanines [44]. The energy of such resonant Raman scattering is significantly less susceptible to electric field-driven shifts, since it measures a difference between vibrational levels, which experience basically the same shift, cancelling each other effectively. Based on this and the fact that these features are dominant only in the region from 1.326 to 1.330 eV with the 785 nm laser, they can be distinguished from the TEPL.

**Figure 2:**
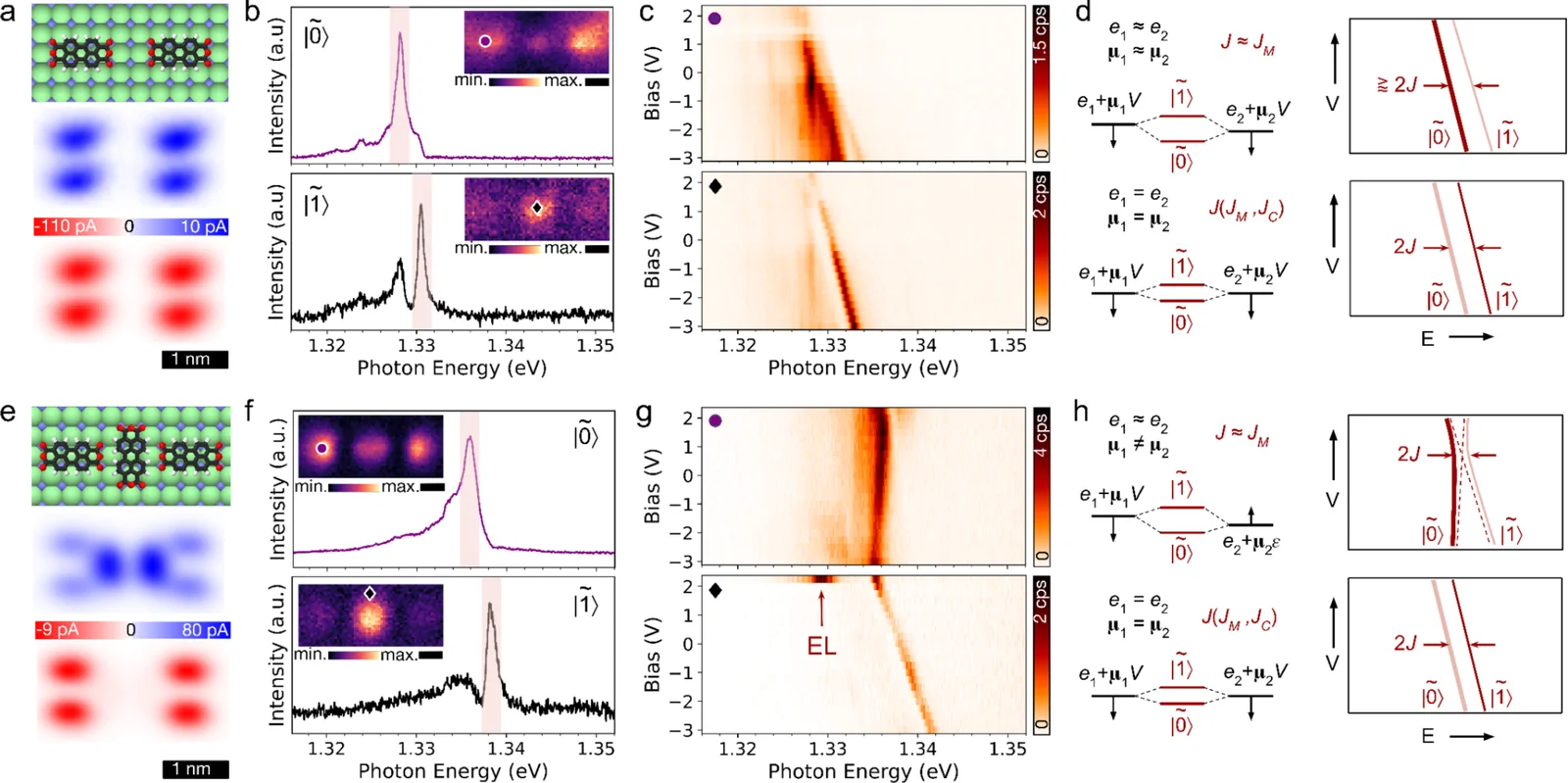
(a) Model of a PTCDA dimer on NaCl and constant-height current images taken at 1.0 and −0.8 V, size 3.8 × 2.0 nm^2^. (Cl^−^ and Na^+^ atoms are marked green and purple, respectively). (b) TEPL point spectra of the dimer at 0 V, acquired at the peripheral and central sites (marked by a purple dot and a black diamond, respectively). The insets show TEPL intensity maps for 1.328 and 1.331 eV, respectively, measured at constant height at 0 V, with size 6.0 × 2.6 nm^2^, 60 × 26 points. (c) Bias-dependent TEPL spectral intensity represented as heatmaps for the sites marked in (b). (d) Schematic diagram for the on-site energies (*e*_1,2_) and eigenenergy levels (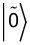, 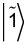) and linear Stark shifts (μ_1,2_) for rationalization of the bias-induced shifts of the collective excitonic modes in the PTCDA dimer, based on the direct and nanocavity-mediated interexcitonic coupling (*J*_M_ and *J*_C_, respectively). (e) Model of PTCDA trimer aggregate and constant-height images at ±1 V with size 3.8 × 2.0 nm^2^. (f) TEPL point spectra acquired at the periphery and center region of the trimer at 0 V. The insets show TEPL signal maps for 1.336 and 1.338 eV, respectively, measured at constant height at 0 V, with size 6.0 × 2.6 nm^2^, 60 × 26 points. (g) Bias-dependent TEPL spectral intensity heatmap at the sites marked in (f). The arrow denotes the electroluminescence onset of the middle molecule at 2.25 V. (h) Schematic diagrams for the on-site energies (*e*_1,2_) and eigenenergy levels (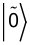, 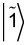) and linear Stark shifts (μ_1,2_) for rationalization of the bias-induced divergent shifts of the collective excitonic modes for the PTCDA trimer at various measurement positions.

Next, to investigate the photophysical properties of PTCDA anion aggregates, we created the simplest model systems, that is, dimers. It was achieved with molecular manipulation [34], arranging two molecules to be on the same Cl^−^ row along the ⟨110⟩ direction on the NaCl(001) surface, with their longer axes aligned collinearly. The measured intermolecular center-to-center distance of 1.95 nm corresponds to 5 Cl^−^ lattice spacings, as shown in Figure 2a. The zero-bias TEPL spectrum measured at the peripheral end of either molecule of the dimer has a peak at 1.328 eV, nearly identical in shape to that of a single molecule. However, the TEPL intensity spatial distribution at this energy over the dimer (inset of Figure 2b) does not correspond to a simple superposition of the individual molecular emission patterns. Instead, the strongest intensity is localized at the outer sides of the molecules, and the inner region between them exhibits a considerably weaker contribution. Furthermore, the spectrum obtained in the central region, in addition to the 1.328 eV peak, has a sharp feature emerging at 1.331 eV. The TEPL map at this energy shows higher intensity between the two molecules. This is an expected behavior in two identical coulombically coupled excitons with a dominant dipolar character, forming a prototypical J-aggregate [45]. We show the corresponding characteristic simulated TEPL patterns in Figure S1 (Supporting Information File 1). Therefore, we can attribute the two bands at 1.328 and 1.331 eV to the emission from the superradiant (bright) and subradiant (dark) modes of the coupled D_0_^−^ ↔ D_1_^−^ excitons of the molecules, which we denote as 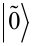 and 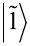, respectively.

The bias dependence of the 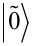 emission measured at the dimer periphery (Figure 2c) (purple circle) closely resembles that of the single molecule, including energy shift and restructuring. In contrast, the lineshape of 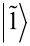 taken in the center of the dimer (black diamond) is considerably sharper in the entire bias range, with the peak energy position following a linear trend (approximately −0.8 meV/V). Similar to Figure 2b, where the spectrum of the 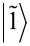 contains a hint of the 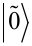 state, the bias-dependent spectra show a mixture of the two states, too. This is a result of a nonzero nanocavity coupling with the 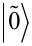 state, regardless of the measurement position above the aggregate. When the distance of the parallel molecules in the dimer is varied, again by manipulation or using thermal self-assembly (see Figure S2 and Figure S3, Supporting Information File 1), the near correspondence of the 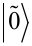 and 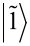 Stark shifts is preserved; only the energy splitting narrows as the distance is increased, due to a diminishing coupling among the two excited states. The linear response of both excitonic modes to the applied electric field can be rationalized as a result of the simultaneous shifting of the on-site energies of both chromophores, as illustrated in Figure 2d and discussed further below. The recent theoretical work on Stark shifts on single molecules in nanocavities [12] implies that the Stark shifts of individual PTCDA molecules, which are centrosymmetric and lack a static dipole, should originate from the perturbation caused by the inhomogeneous electric field in the junction.

In a similar system (assembled by thermal diffusion), where a third, interstitial PTCDA is wedged between the two chromophores in a perpendicular orientation and stabilized by the carboxylic group–hydrogen interactions (see Figure 2e), we observe a specific change of the apparent Stark shift for the dominating peak measured at the periphery. The zero-bias TEPL spectra in Figure 2f at the peripheral and the central sites of such a symmetrical trimer show two peaks at 1.336 and 1.338 eV, respectively. In this symmetrical trimer, the current maps and d*I*/d*V* (Figure S4c, Supporting Information File 1) indicate that the central PTCDA unit maintains an equilibrium neutral charge, in accord with our previous findings [34]. It shows that the electronic signatures characteristic of tunneling into the half-occupied orbital of the anion appear above the Fermi level, confirming that this orbital is unoccupied at zero bias. Considering that only the peripheral anions couple and contribute to the collective states, resolved in the TEPL intensity distribution maps (insets of Figure 2f), we can attribute the peaks again to the 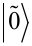 and 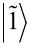 states. However, the bias dependence of the 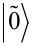 emission measured at the periphery is different from the case of the dimer; the lineshape is sharper and more uniform across the biases, with a reversed Stark shift of approximately 0.2 meV/V and a trend change above 1.5 V. The 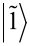 emission is very similar to the case of dimers, sharp and shifting with a similar magnitude (−1.2 meV/V). At biases above 2.0 V, where a reversible switch of the central neutral molecule to an anion is expected, we observe its electroluminescence appearing at about 1.329 eV (denoted by a red arrow in Figure 2g).

**Figure 3:**
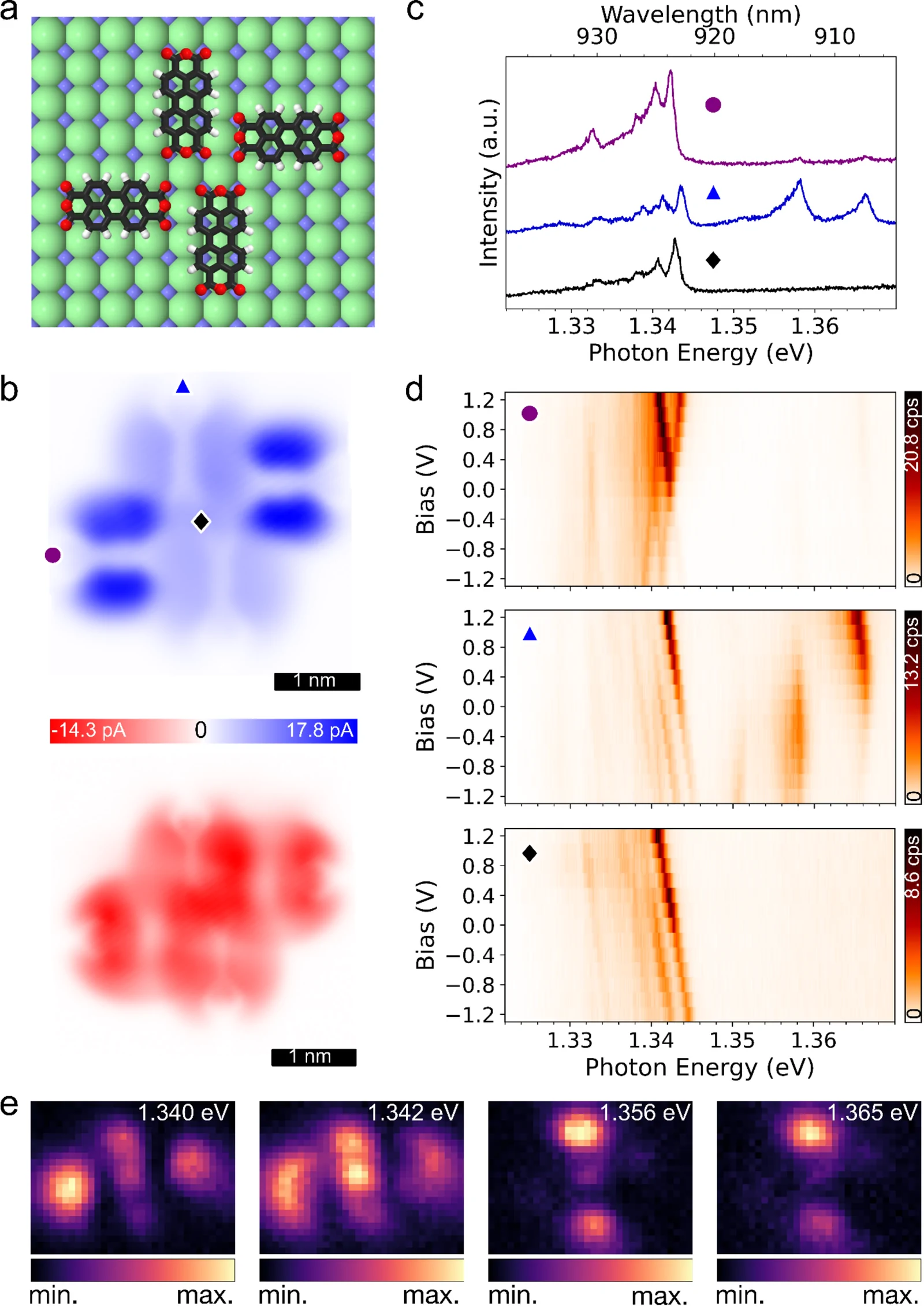
(a) Model of PTCDA tetramer on NaCl (Cl^−^ and Na^+^ atoms are marked green and purple, respectively). (b) Constant-height current images taken at +0.5 V and −0.25 V, 5 pA, size 3.56 × 3.56 nm^2^. (c) TEPL point spectra of the tetramer at 0 V, acquired at the two peripheral and the central positions (denoted by purple dot, blue triangle, and black diamond, respectively, also in (b)). (d) Bias-dependent TEPL spectral intensity represented as heatmaps for the sites marked in (b). (e) TEPL intensity maps for 1.340, 1.342, 1.356, and 1.365 eV, measured at constant height at 0 V, with size 6.0 × 2.6 nm^2^, 60 × 26 points.

We extend the investigation to larger and more complex aggregates, here a particular tetramer with a twofold rotationally-symmetrical (*C*_2_) chiral structure (Figure 3a). It comprises two pairs of equivalent molecules due to the symmetry of the arrangement, one pair that is oriented along the longer dimension of the cluster and one pair oriented along the shorter dimension. The current maps and d*I*/d*V* spectra (Figure 3b and Figure S4d, Supporting Information File 1, respectively) show that the electronic signatures of the two types of molecules in the cluster are shifted in energy compared to monomers and the peripheral molecules in the trimers, but their general characteristics still correspond to those of PTCDA anions. The electronic signature of the pair along the shorter dimension of the cluster is shifted to slightly higher energies with respect to the pair oriented along the longer dimension.

The TEPL spectra at zero bias in Figure 3c, measured at three different representative locations above the tetramer (shown in Figure 3b), display multiple peaks that evolve in analogy to the dimers and trimers (Figure 3d). At the center point of the tetramer (black diamond), the bias dependence shows a dominating sharp spectral feature (1.345 eV at −1.2 V) with a pronounced negative Stark shift (−1.6 meV/V), accompanied by weaker sidebands at ≈2.5 and ≈5 meV lower energies. When measured over one of the equivalent peripheral molecules farther from the center (denoted with a purple circle), these sidebands seem to switch the polarity of the Stark shift (1.2 meV/V), resulting in an apparent crossover with the dominant spectral feature. The overlap is observed near zero bias and is consistent with the photon map (Figure 3e) that shows a hybrid pattern of intensity localized at the periphery and in the aggregate center at the same time. Therefore, the dominating band and its sideband at ≈2.5meV lower energy, measured in the tetramer center position, can be assigned to the in-phase and out-of-phase fingerprints of the excitonic coupling among the two molecules oriented along the larger dimension of the tetramer. Finally, when measuring at the periphery, in the direction of the shorter dimension (marked with blue triangles), broad peaks appear at higher energies above 1.35 eV, which we therefore assign to a dominant coupling among the two molecules oriented along the shorter dimension of the tetramer, again based on the spatial correlation with the respective photon map in Figure 3e. In general, hallmarks of a similar behavior, including the change of the Stark shifts of spectral features when measured at the center and periphery of the aggregate, are also observed in more complex aggregates (shown in Figure S5, Supporting Information File 1), although the spectral manifestations of the collective modes are less clear, most likely due to the higher amount of PTCDA units involved, coupling strength constrained by the geometry and the limited resolution of the method.

The unifying trait of all the described structures is a fairly consistent proportional bias-induced shift of the dark mode between −1.2 and −1.6 meV/V, regardless of the nanocavity position. The divergent and sharpened bright modes of the trimer, tetramer, and hexamer were observed with the nanocavity positioned above their respective peripheral parts, whereas no such effect was present on the simple dimers. The specific aspect of the dimers, compared to the other studied aggregates, is the absence of the interstitial PTCDA units between the peripheral molecules. For an elementary phenomenological rationalization of the observed peak shifts in the dimer in Figure 2c, we consider two electronic transitions with intrinsic on-site energies *e*_1_ and *e*_2_, coupled through the effective overall coupling *J* (which is a nontrivial product of the interexciton coupling *J*_M_ and an additional coupling mediated by the nanocavity *J*_C_) and shifted by the applied bias *V* with proportional factors μ_1_ and μ_2_, expressed as *E*_1,2_ = *e*_1,2_ + *V*μ_1,2_. The resulting eigenenergies will be simply given by [6,46–47]:


[1] $$E_{\pm }=\frac{E_{1}+E_{2}}{2}\pm \sqrt{{\left(\frac{E_{1}-E_{2}}{2}\right)}^{2}+J^{2}}$$

For the situation with a nanocavity symmetrically placed above the center of the dimer, the intrinsic on-site energies should be equal, that is, *e*_1_ = *e*_2_. The observed parallel shifting of the 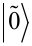 and 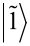 modes with bias can then be understood as a result of a similar effect of the electric field on the two sites, regardless of the nanocavity position (for simplicity μ_1_ = μ_2_). Moving the nanocavity above one of the sites results in a small change among the respective constant Lamb and Stark shifts (i.e., *e*_1_ ≈ *e*_2_ and μ_1_ ≈ μ_2_) and a reduced effective coupling *J* due to the diminishing role of the nanocavity-mediated part *J*_C_. The consequence would be a slightly smaller separation of the 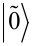 and 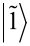 modes and a small deviation from their parallel evolution, as shown in the diagram in Figure 2d.

In contrast, to use the same model to reconcile the diverging shift of the 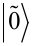 mode of the trimer with a nanocavity positioned at the periphery (Figure 2g), the bias-dependent on-site energies of the peripheral chromophores need to respond to the field differently. Specifically, one of the coefficients μ_1_ and μ_2_ must be strongly altered to reproduce the observed bias dependence of the 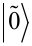 state eigenenergy within the bias window (see Figure 2h). This scenario would result in an avoided crossing at the point of the two on-site energy equality *E*_1_ = *E*_2_. Despite a lack of signal for the 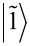 state in Figure 2g, a hint of the avoided crossing onset is visible around 1.8 V. We have attempted to optimize the parameters of this simple model to match the observed dependencies of the mode energies. The results are plotted with a good match over the data in Figure S6 (Supporting Information File 1), and the corresponding parameters are included in Table S1 (Supporting Information File 1). Since the divergent Stark shifts are observed for the modes at the peripheries in the trimer, tetramer, and hexamer (in contrast to the simpler dimers), we propose that this behavior primarily originates from a specific electrostatic screening effect at the perpendicularly oriented molecules between the coupled PTCDA units, regardless of their charge state.

On the basis of this simple model, we can also get an elementary understanding of the behavior of the collective modes in the tetramer cluster. The parallel bias dependence of the bright and dark modes measured at the center can again be understood as a result of their equally shifting on-site energies. Conversely, with the nanocavity position above one of the molecules, the crossover-like appearance can be seen as stemming from the diverging on-site energies. The broad spectral features above 1.35 eV associated with the pair of molecules closer to the tetramer center indicate that the corresponding on-site energies are relatively strongly increased, most likely due to interactions with the surrounding PTCDA units. In the larger radical excitonic clusters represented in this work by the tetramer and hexamer, it should be considered that the individual units are switching between various charges (as the neutral and anion are close in energy), which could give rise to the observed additional multiplicity of the bands. However, further measurement, analysis, and simulations would be needed to fully corroborate this.

## Conclusion

Using TEPL, we have achieved the direct and local electric-field tuning of the excitonic eigenstates of selected coulombically coupled PTCDA anion chromophore aggregates. A consistent, nearly linear Stark shifting of the delocalized states was found in several symmetrical clusters of various sizes in response to the electric field applied within the nanocavity gap between the tip and the sample. In clusters containing interstitial molecules, the nanocavity located above the high-symmetry points of the clusters produces very similar energy shifts for the dark and bright states, whereas measurements in off-symmetry peripheral positions lead to diverging shifts of the bright states. This phenomenon can be reconciled by considering a specific renormalization of the on-site Stark shift coefficients in a simple coupled-exciton model and extrapolating to the more complex aggregates. We also revealed a general sharpening of the excitonic modes with increasing cluster size. Overall, our findings highlight the important role of the nanocavity localization in the interexciton coupling and on-site Stark shifts (in particular, for the bright modes). This study also shows that tailored radical aggregates in plasmonic nanocavities can serve as templates for developing controllable excitonic systems.

## Experimental

Experiments were performed in an ultrahigh vacuum environment using a low-temperature STM (Createc GmbH) operating at 6 K. The Ag(111) single crystal was prepared by standard cycles of Ar^+^ ion sputtering and thermal annealing. For the decoupling layers, NaCl was thermally evaporated from a custom-built evaporator at 607 °C onto the half-covered Ag sample maintained at 114 °C for 4 min, resulting in the formation of a partial mixture of 2–4 monolayers (MLs) of NaCl islands. PTCDA molecules were deposited onto the NaCl/Ag(111) surface within the STM head for 3 min at 320 °C. After deposition, the sample was left to thermalize for 1–2 min (estimated temperature 100–150 K) in order to facilitate thermal diffusion and the formation of molecular aggregates. For dimer formation, the molecules were manipulated onto the same Cl^−^ row of the NaCl surface, with their transition dipoles aligned collinearly. The tip was approached to the H-terminated region of the molecule opposite to the intended movement with 3 V and a 20 pA feedback setpoint. The feedback loop was then turned off, and bias and setpoint were changed to 1.5 V and 5 pA. With these conditions, the feedback was turned back on, and the area was rescanned to inspect the outcome. The procedure was repeated until the molecule was moved to the desired position.

A plasmonic Ag tip was conditioned by applying voltage pulses and controlled indentations on a clean Ag(111) surface and tuned to cover both excitation and emission energy ranges. The excitation source was a single-frequency continuous-wave infrared laser (785 nm). The laser, with a total output power of 1.1 mW and polarization plane aligned with the tip–sample axis, was focused into the tunneling junction by an aspheric lens through a confocal optical setup, employed also for the photoluminescence detection previously [14,38,48]. To acquire the photon rate, a single-photon avalanche photodiode from Perkin-Elmer (SPCM-AQR-15) was used. The TEPL spectra were acquired with an Andor Kymera 328i spectrograph equipped with a Newton 920 CCD detector with a 1200 groove/mm, 500 nm blaze grating, providing a 60 nm spectral range and 0.25 nm resolution (approximately 0.4 meV at 933 nm). The acquisition time was typically 30 s per spectrum for the point spectroscopies and 1–2 s for the TEPL maps, with all measurements performed in constant-height mode. All TEPL intensity maps were integrated over a ±1 meV energy window range. The tip height was set for both TEPL point measurements and maps as follows: With feedback on and a setpoint of 1.2 V and 5 pA, the tip was positioned over the brightest lobe of the molecule/aggregate, and the feedback was switched off. Subsequently, the tip was brought to the particular measurement locations for point spectroscopy or mapping. The differential conductance (d*I*/d*V*) spectra were measured using a standard lock-in approach with a modulation amplitude of 20 mV.

For fitting, we tracked the peak maxima directly from the spectra after background and cosmic ray removal and used linear regression to fit the trends and evaluate the errors with 95% confidence intervals. We had to exclude the voltage and energy ranges where the suspected Raman resonances dominated and where the maxima were ambiguous; for example, for the monomers and the peripheral site of the dimer, we excluded the bias range of [−1.7,0] V. For the center site of the dimer, we excluded [1.5,1.8] V, and for the peripheral site of the trimer, [2.1,2.5] V. Finally, for the center site of the tetramer, we defined a narrow, shifting band to capture the position of the strongest peak. The fitting parameters, ranges, and error bars are summarized in Table S2 (Supporting Information File 1) and plotted in Figure S7 and Figure S8 (Supporting Information File 1). We have repeated the Stark shift measurements on monomers and trimers with different tips, obtaining very similar energy shifts as presented here.

## Supporting Information

File 1 Additional experimental data.

## Data Availability

All data that supports the findings of this study is available in the published article and/or the supporting information of this article.
