# Supplementary material for: Locally induced Stark shifts of collective excitonic modes in polyradical aggregates
Source: Beilstein J Nanotechnol. 2026 Aug 25;17:1185–93. doi: 10.3762/bjnano.17.81 (PMC13550844; doi:10.3762/bjnano.17.81)
Supplement: Supplementary file 1 [file Beilstein_J_Nanotechnol-17-1185-s001.pdf]

BEILSTEIN JOURNAL OF NANOTECHNOLOGY

Supporting Information

for

Locally induced Stark shifts of collective excitonic modes in polyradical aggregates

Amandeep Sagwal, Rodrigo Cezar de Campos Ferreira, Petr Kahan, Maximilian Rödel, Jindřich Nejedlý, Jiří Doležal and Martin Švec

Beilstein J. Nanotechnol. **2026,** *17,* 1185–1193. doi:10.3762/bjnano.17.81

Additional experimental data

License and Terms: This is a supporting information file under the terms of the Creative Commons Attribution License (https://creativecommons.org/licenses/by/4.0). Please note that the reuse, redistribution and reproduction in particular requires that the author(s) and source are credited and that individual graphics may be subject to special legal provisions.
The license is subject to the *Beilstein Journal of Nanotechnology* terms and conditions: (https://www.beilstein-journals.org/bjnano/terms)

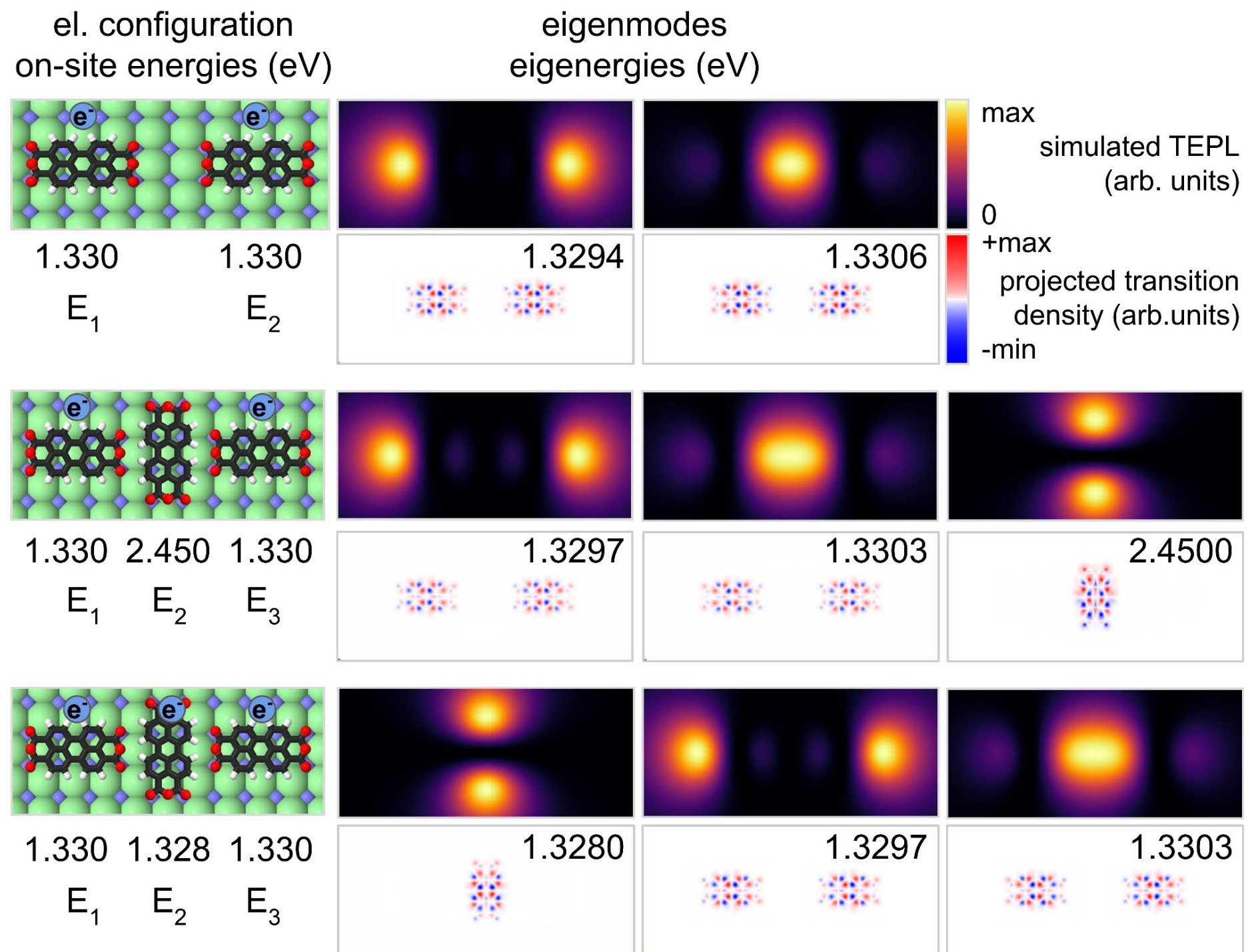

Figure S1: Zero-field collective excitonic modes calculation and the corresponding TEPL pattern simulations. $E_{1\text{–}3}$ are the first-excited-state on-site energies for individual chromophores. The transition densities and eigenenergies are shown for the eigenmodes of the systems. Transition densities for the anion and neutral first-excited states were used from [1]. The eigenmodes of the systems were determined by solving a Hamiltonian with the on-site energies of individual chromophores in the aggregates on its diagonal. Pairwise coulombic couplings in the off-diagonal terms were evaluated on a discrete grid from precalculated transition densities, taken from our previous publication [1], using the PPR package on GitHub, photonMap branch [2]. For speed and simplicity, the coulombic coupling from the transition densities was taken only in the molecular plane.

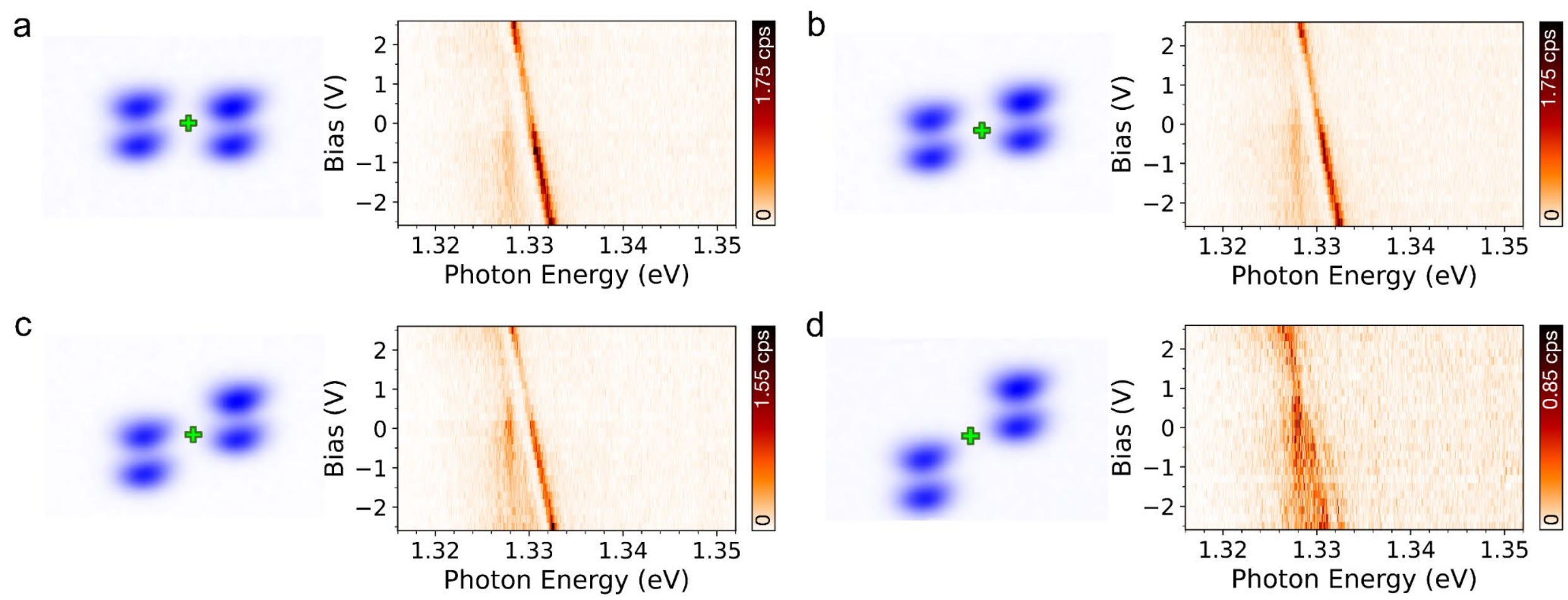

Figure S2: TEPL of parallel PTCDA dimer manipulated to various intermolecular distances. The distance between two parallel oriented PTCDA molecules was varied by moving one of them along the ⟨110⟩ direction on the NaCl(001) surface, resulting in four different geometries, corroborated by the constant-height current imaging at 1.2 V. The bias-dependent TEPL was measured at a center position (marked with green crosses) in order to simultaneously detect the $|\tilde{0}\rangle$ and $|\tilde{1}\rangle$ modes.

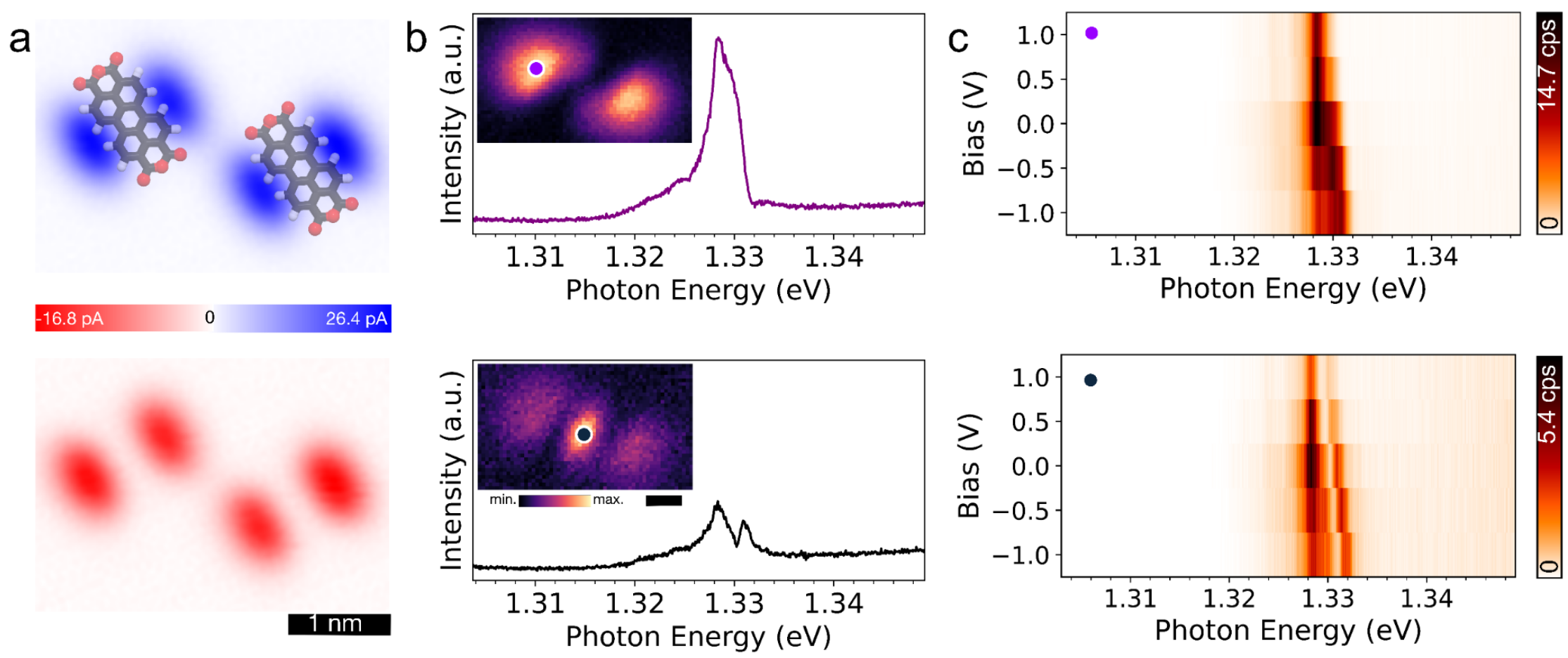

Figure S3: A representative spontaneously formed parallel PTCDA dimer with a different orientation. (a) Constant-height scans of a dimer with ball-and-stick model overlay taken at +1.0 V and −0.7 V, 5 pA, size 3.5 × 2.5 nm^2. (b) TEPL spectra of both $|\tilde{0}\rangle$ and $|\tilde{1}\rangle$ modes taken at 0 V. The insets are the TEPL maps at 1.329 and 1.332 eV, respectively, acquired at 0.5 V, size 6.0 × 3.5 nm^2, 60 × 35 points. (c) Bias-dependent TEPL spectral intensity heatmap acquired at the peripheral and central positions (marked with purple and black dots in the inset of (b), respectively).

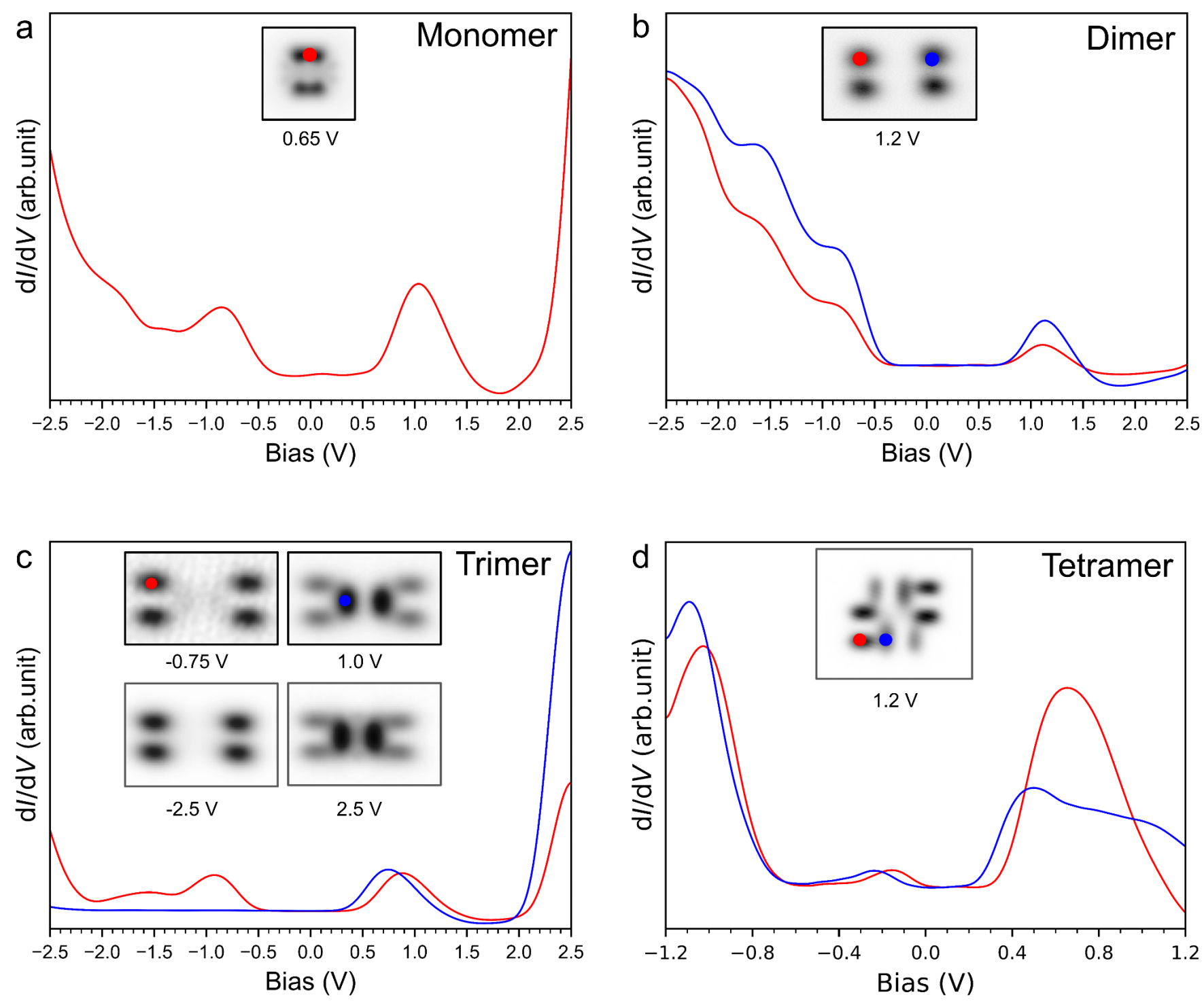

Figure S4: Differential conductance (dI/dV) of aggregates with illumination, taken for (a) monomer, (b) dimer, (c) trimer, and (d) tetramer. Constant-height current images are shown in the insets. The red and blue spectra were acquired at the positions marked by red and blue dots, respectively, in the corresponding inset. The dI/dV spectra were measured using the standard lock-in technique with bias modulation 20 mV and 927 Hz.

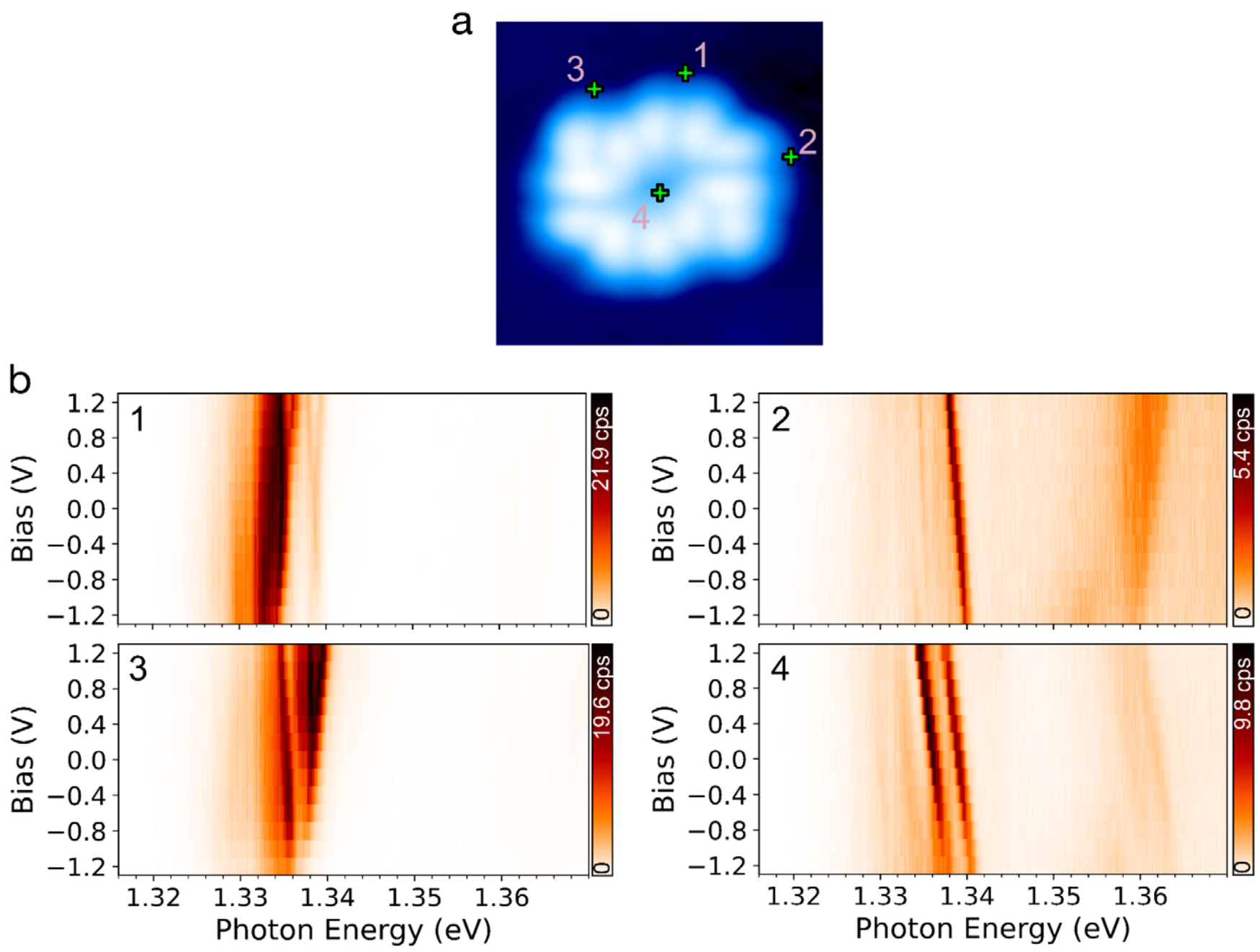

Figure S5: Hexamer PTCDA aggregates. (a) STM constant-current topography of a hexamer aggregate formed by thermal diffusion, with marked positions (1–4) of bias-dependent TEPL measurements. Scanning conditions: 1.2 V, 4 pA, size: 7 × 7 nm^2 (b) Bias-dependent TEPL heatmaps measured at positions marked in (a) at 0 V.

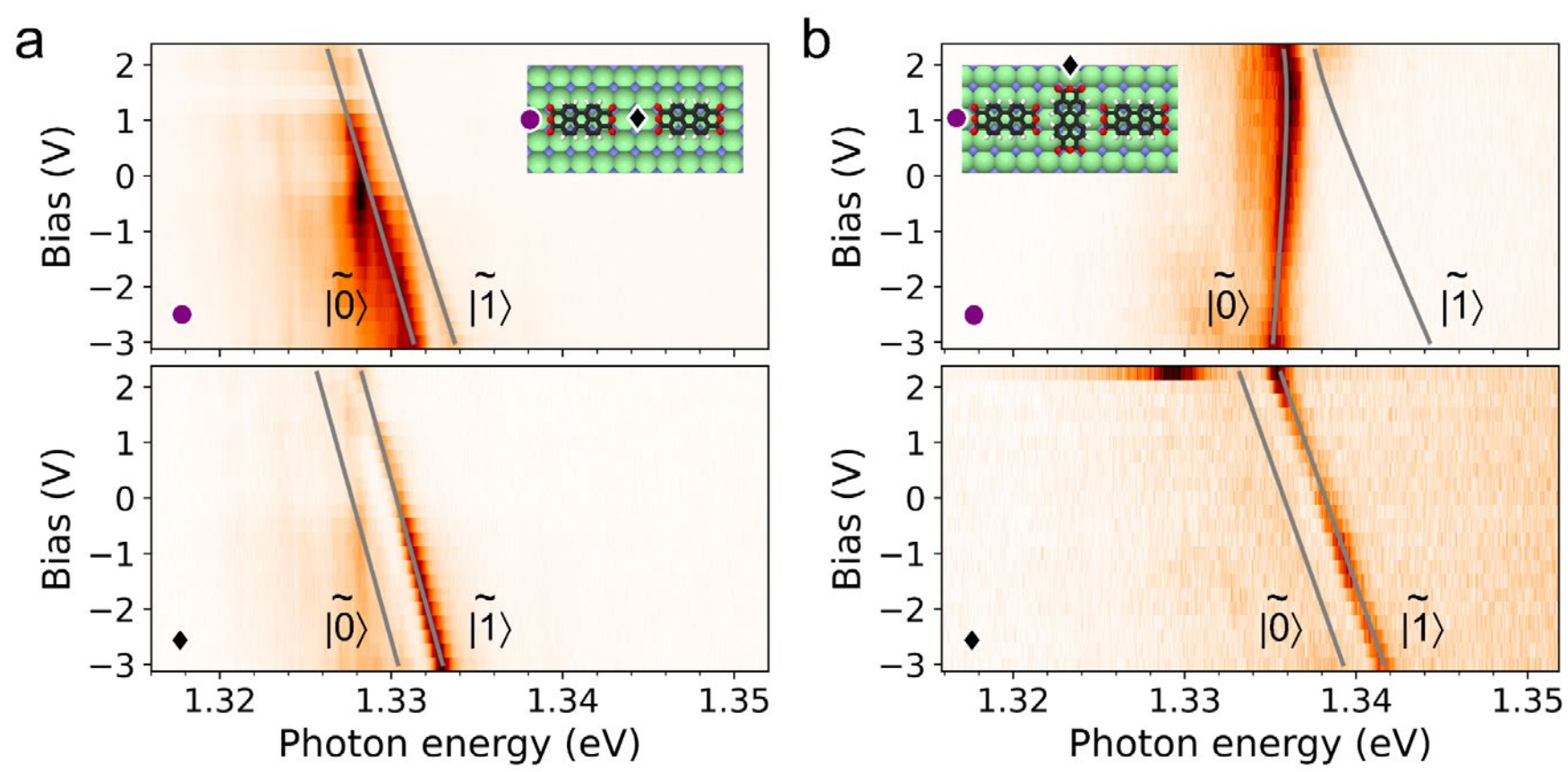

Figure S6: Matching the two-exciton model to bias-dependent TEPL for (a) the dimer and (b) the trimer. The trends of the bias-dependent peak energy positions for $|\tilde{0}\rangle$ (purple dot) and $|\tilde{1}\rangle$ (black diamond) modes were generated with the parameters of the model in Table S1. The insets show the ball-and-stick models of the aggregates.

Table S1: Parameters of the two-exciton model, used for the energy trends overlay in Figure S6. The positions (sites) are according to Figure 2.

**Aggregate**	**Site**	e_1 **(eV)**	e_2 **(eV)**	μ_1 **(meV/V)**	μ_2 **(meV/V)**	J **(meV)**
dimer	center	1.329	1.329	−0.90	−0.90	1.30
dimer	periphery	1.330	1.329	−1.10	−0.90	0.90
trimer	center	1.337	1.337	−1.20	−1.20	1.20
trimer	periphery	1.340	1.336	−1.40	+0.25	0.90

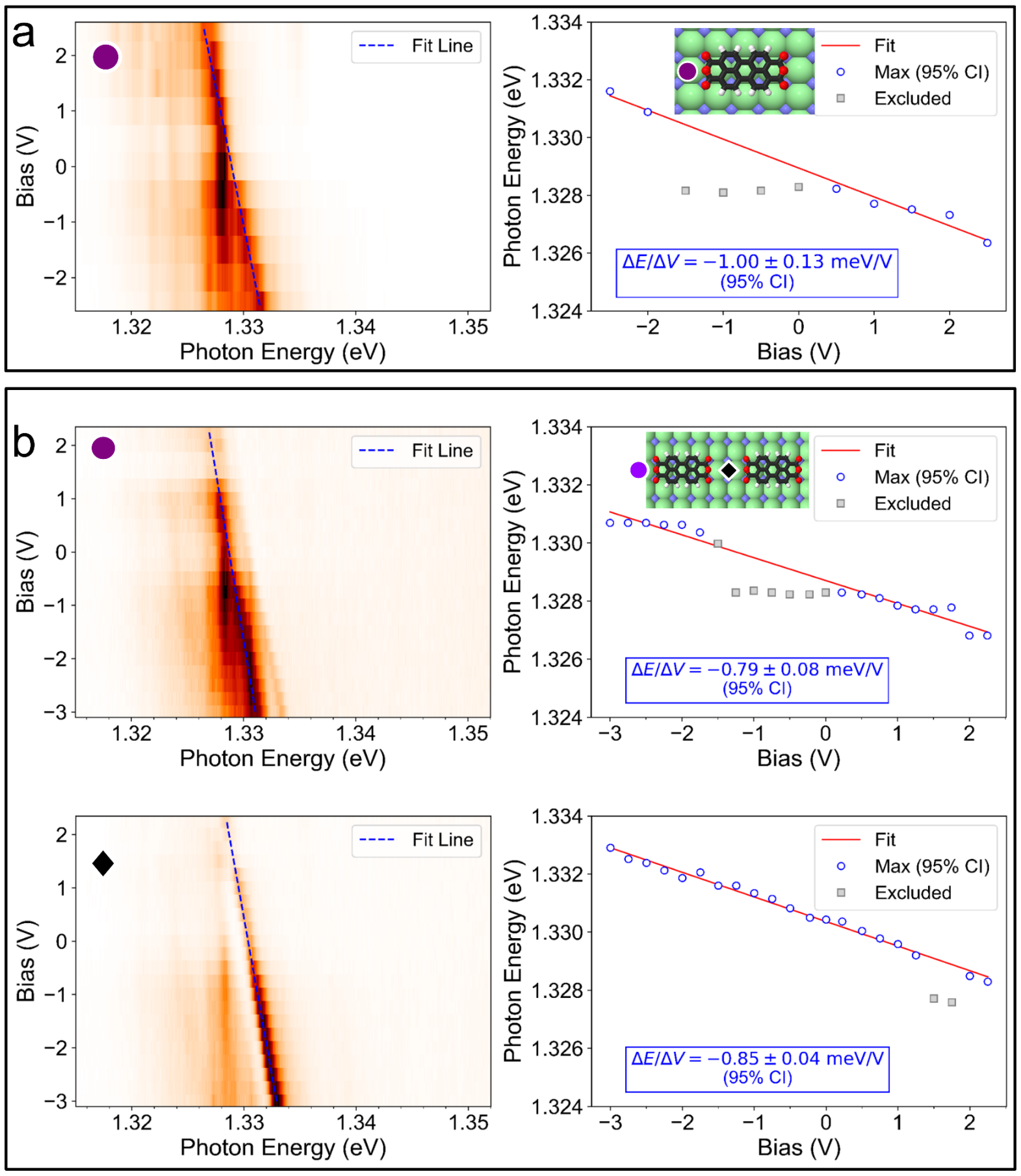

Figure S7: Fitting of the Stark shifts for (a) monomer and (b) dimers. The insets show the ball-and-stick models of the molecule and its aggregates. The measurement positions (sites) correspond to Figures 1–2.

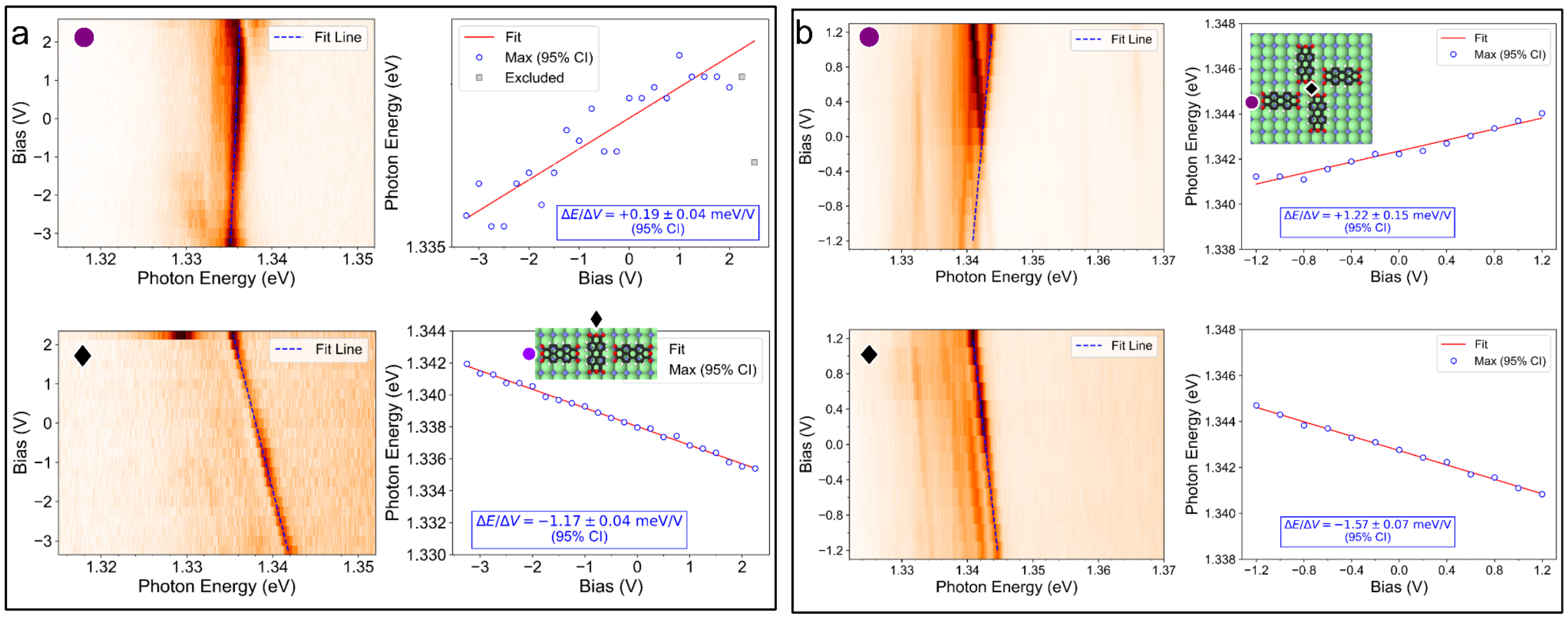

Figure S8: Fitting of the Stark shifts for (a) trimers and (b) tetramers. The insets show the ball-and-stick models of the molecule and its aggregates. The measurement positions (sites) correspond to Figures 2–3.

Table S2: Summary of fitted Stark shift trends, fitting ranges, and errors across monomer, dimers, trimers, and tetramers datasets with the positions (sites) corresponding to Figures S7 and S8.

**System**	**Site**	**Bias range (V)**	**Fitted bias range (V)**	**Stark shift $\Delta E/\Delta V$ (meV/V)**	**Zero-bias energy (eV)**
monomer	periphery	[−2.50, 2.50]	[−2.50, −2.00]; [0.50, 2.50]	−1.00 ± 0.13	1.328947
dimer	center	[−3.00, 2.25]	[−3.00, −1.25]; [2.00, 2.25]	−0.85 ± 0.04	1.330367
dimer	periphery	[−3.00, 2.25]	[−3.00, −1.75]; [0.25, 2.25]	−0.79 ± 0.08	1.328709
trimer	center	[−3.25, 2.50]	[−3.25, 2.50]	−1.17 ± 0.04	1.338017
trimer	periphery	[−3.25, 2.50]	[−3.25, 2.00]	+0.19 ± 0.04	1.335804
tetramer	center	[−1.20, 1.20]	2 meV band, start: −1.3445 eV at 1.2 V, end: −1.3400 eV at +1.2 V	−1.57 ± 0.07	1.342732
tetramer	periphery (longer dimension of the cluster)	[−1.20, 1.20]	2 meV band, start: −1.342 eV at -1.2 V, end: −1.346 eV at 1.2 V	+1.22 ± 0.15	1.342358

References

[1] Doležal, J.; Canola, S.; Hapala, P.; de Campos Ferreira, R. C.; Merino, P.; Švec, M. *ACS Nano* **2022,** *16,* 1082–1088. doi:10.1021/acsnano.1c08816

[2] GitHub - Probe-Particle/ppafm: Classical force field model for simulating atomic force microscopy images. · GitHub. https://github.com/ProkopHapala/ProbeParticleModel (accessed Aug 19, 2026).
